# An Evaluation of Skin and Immunological Responses after Using a Novel Cross-Linked Porcine-Based Dermal Injectable Collagen with Lidocaine for Nasolabial Fold Correction

**DOI:** 10.3390/jcm13175241

**Published:** 2024-09-04

**Authors:** Hao-Chih Tai, Yi-Hua Liao, Ya-Ching Chang, Chin-Yi Yang, Shyue-Yih Horng, Yuan-Sung Kuo, Yi-Shuan Sheen, Yu-Huei Huang, Rosaline Chung-Yee Hui, Tim-Mo Chen, Yuan-Sheng Tzeng, Chih-Hsin Wang, Shou-Cheng Teng, Chun-Kai Oscar Chang, Chang-Yi Chou

**Affiliations:** 1 Department of Surgery, National Taiwan University Hospital and National Taiwan University College of Medicine, Taipei 100, Taiwan; 2 Department of Dermatology, National Taiwan University Hospital and National Taiwan University College of Medicine, Taipei 100, Taiwan; 3 Department of Dermatology, Linkou Chang-Gung Memorial Hospital, Taoyuan 333, Taiwan; 4 Department of Dermatology, New Taipei Municipal TuCheng Hospital, New Taipei City 236, Taiwan; 5 Department of Dermatology, Chang-Gung Memorial Hospital, Keelung 204, Taiwan; 6 Division of Plastic Surgery, Tri-Service General Hospital, Taipei 100, Taiwan

**Keywords:** bovine collagen, filler, lidocaine, medical devices, nasolabial fold, porcine collagen

## Abstract

**Background:** Hypersensitivity to the new dermal injectable porcine-based collagen with lidocaine featuring a novel cross-linking technology (test filler) for nasolabial fold correction was compared to the commercially available traditional cross-linked dermal injectable porcine-based collagen with lidocaine (control filler). **Methods:** Recruited participants (n = 279) received a single 0.1 mL intradermal injection of either test filler or control filler in the left forearm as a screening skin allergy test. Injection sites were assessed clinically at 24 h post-implant. Treatment was given to 252 successfully screened participants, and injection sites were monitored for 21 days. Immunological examinations were performed at screening and then at 4 and 24 weeks post-treatment. Observations for adverse events continued until the 52nd week. **Results:** Intradermal allergy testing results were negative for all the test recipients (0/124) and positive for two control recipients (2/132, 1.5%). Most of the participants exhibited no changes in serum immunoglobulin (IgG, IgM) and complement (C3, C4) levels. No serious adverse events related to the device were recorded. Most adverse events were common complications of dermal filler treatment and were related to the injection site. Most adverse effects were resolved or under control by 52 weeks. **Conclusions:** Hypersensitivity reactions with the test filler were lower than those with the control filler, validating the safe use of test filler for nasolabial fold correction without the need for pretreatment skin testing.

## 1. Introduction

Advancements in biomaterials and injection technology have fueled the rapid growth of minimally invasive cosmetic surgery and increased the demand for dermal fillers. Injecting dermal filler into the mid or deep dermis can improve the appearance of moderate-to-severe wrinkles or folds, creating more youthful-looking skin. The introduction of bovine collagen dermal fillers in the early 1980s has led to the widespread use of soft tissue dermal fillers in facial cosmetic surgery. The favorable attributes of collagen (biocompatibility, low immunogenicity, cell interaction, mechanical strength, moisture retention, biodegradability, and the potential for mass production) make this material ideal for facial soft tissue repair.

In 1981, the United States Food and Drug Administration (U.S. FDA) approved the first dermal filler for the correction of facial wrinkles, Zyderm I^®^ (Allergan, Inc., Goleta, CA, USA) [1,2], a purified suspension of 3.5% bovine collagen for injection into the dermis using a 30- or 32-guage needle [3]. Subsequently, Zyderm II^®^ (Allergan, Inc., Goleta, CA, USA) containing 6.5% bovine dermal collagen for injection into the middle layer of the dermis was granted U.S. FDA approval in 1983 for the correction of coarser glabellar wrinkles, nasolabial folds, and acne scars [1,2]. In 1985, Zyplast^®^ (Allergan, Inc., Goleta, CA, USA), containing 3.5% bovine dermal collagen cross-linked with 0.0075% glutaraldehyde to strengthen molecular bonding, received FDA approval for deep dermal injection [1,2]. Widespread immunogenicity and hypersensitivity reactions to bovine collagen fillers led to the recommendation of two consecutive skin tests (at 2 and 4 weeks) prior to treatment, with positive results excluding their use [2]. In 2010, Zyderm^®^ and Zyplast^®^ were discontinued by Allergan, Inc, in response to patients’ reluctance to undergo time-consuming skin allergy tests.

In 2003, the U.S. FDA approved the injectable dermal fillers CosmoDerm I^®^, CosmoDerm II^®^, and CosmoPlast^®^, which are cultured from human fibroblasts for soft tissue filling, with therapeutic effects lasting for 3–4 months [1,2]. The strengths and injectable properties of these products are similar to those of Zyderm I^®^, Zyderm II^®^, and Zyplast^®^, respectively. CosmoDerm I^®^ has a collagen content of 35 mg/mL and is used for treating fine wrinkles and epidermal imperfections, whereas CosmoDerm II^®^ has a collagen content of 65 mg/mL and is suitable for moderate deficiencies. CosmoPlast^®^, a cross-linked human collagen with glutaraldehyde, also has a collagen content of 35 mg/mL and is used for filling coarser wrinkles at the deep dermis level, similar to the indications for Zyplast^®^ treatment. These products are sourced from cell-cultured fibroblasts and produced by tissue engineering technology to prevent contamination. Allergic reaction evaluations conducted by the U.S. FDA revealed a very low incidence rate (1.3%) of allergic reactions compared with rates for Zyderm^®^ (3%) [4] and Zyplast^®^, eliminating the need for pretreatment skin allergy tests [5]. In 2008, the U.S. FDA approved Dermicol-P35 (EVOLENCE^®^, Colbar Lifescience LTD, Herzliya, Israel), a porcine collagen dermal filler consisting of a three-dimensional gel network structure of type I porcine collagen [6]. EVOLENCE^®^ is used for medium-to-deep facial wrinkles, such as nasolabial folds, and not only offers long-lasting results (up to 12 months) but also eliminates the need for skin allergy testing. In a 2007 study, statistical analysis predicted that only 0.58% of participants injected with 0.1 mL of intradermal EVOLENCE^®^ would be likely to experience a moderate-to-severe erythematous reaction [7]. In another clinical trial, 61 patients with mild-to-severe nasolabial folds were randomized to receive a porcine-derived collagen filler (TheraFill^®^) and a bovine-derived collagen filler (KOKEN^®^) on contralateral sides of the face [8]. At the 12-month follow-up, comparable efficacy and safety for both collagen products led to the conclusion that porcine-derived collagen is a viable alternative to bovine collagen filler [8].

Dermal filler injections are associated with adverse events that can occur immediately or at a later date. Typically, hypersensitivity reactions occur within 2–3 days after injection, but may also arise several weeks, months, or even years post-injection [9]. Common device-related adverse events include bruising, redness, swelling, pain, itching, and rash. Less common events include the formation of lumps or granulomas, infections, open wounds, sores at the injection site, allergic reactions, and tissue necrosis [9,10,11,12,13].

In 2014, Sunmax Biotechnology (Tainan City, Taiwan) received TFDA approval for the launch of the FACIALGAIN collagen implant (with lidocaine), marking their third approval for collagen implants. No serious device-related adverse events have been reported since the product’s launch in 2014, when it has been used according to instructions. In a post-marketing clinical trial sponsored by Sunmax Biotechnology in 2015, FACIALGAIN demonstrated superior efficacy and safety compared to Sunmax Collagen Implant I-Plus in the correction of nasolabial folds. The post-marketing data revealed an absence of systemic device-related adverse events with FACIALGAIN. Moreover, most device-related adverse events were relieved within 3 days after injection, with remaining symptoms improving continuously within 30 days or resolving without any long-term effects. These findings suggest that the product is suitable for individuals who meet the product indications and do not have any contraindications (e.g., patients who are not in a condition for implantation as evaluated by the surgeon; patients with autoimmune diseases, keloids or severe allergies; patients known to be allergic to collagen or other implants previously injected locally; patients known to be allergic to anesthetic, such as lidocaine; and the safety has not been established in children, pregnant woman, or nursing mothers). FACIALGAIN requires storage between 2 °C and 8 °C. A newer generation of collagen implant, FULLSGEN, has now been developed using a patented cross-linked collagen fiber with lidocaine. Improvements in the cross-linking process, rheological properties, and mechanical strength of FULLSGEN suggest that this device will result in even fewer hypersensitivity reactions. Therefore, this clinical trial compared the incidence rates of allergic reactions and immunological responses between the novel dermal injectable collagen FULLSGEN and the older-generation FACIALGAIN device.

## 2. Material and Methods

The data reported in this paper are from a multicenter, randomized, active-controlled, double-blind clinical trial that evaluated the hypersensitivity of two cross-linked porcine collagen implants (FULLSGEN and FACIALGAIN) (Sunmax Biotechnology, Tainan, Taiwan) for dermal contour correction. The study participants were recruited from Tri-Service General Hospital, Linkou Chang-Gung Memorial Hospital and National Taiwan University Hospital.

### 2.1. Investigational Device

Both investigational and comparator devices used in this study were produced by a certified Good Manufacturing Practice (GMP) manufacturer. The collagen material is extracted from skin obtained from specific pathogen-free (SPF) pigs. After undergoing aseptic filtration and chemical cross-linking with glutaraldehyde containing cross-linker, the highly purified cross-linked collagen fibers are suspended in a phosphate buffer solution containing 0.3% lidocaine and then stored at 2–8 °C. The investigational device (FULLSGEN) has undergone extensive preclinical cytotoxicity, hemolysis, genotoxicity, stimulation, sensitization, acute and subchronic toxicity, implantation, sterility, bacterial endotoxin, and pyrogen testing. The test data indicate that the product is suitable for the clinical trial stage. This product is suitable for correcting facial skin defects, such as filling facial wrinkles. FACIALGAIN was used as the comparator device.

### 2.2. Study Design

Prior to treatment with an injectable collagen implant, we performed an intradermal allergy test with Sunmax FACIALGAIN and Sunmax FULLSGEN according to the randomization results (1:1) before participants were included in the present study and we observed their reaction during a 7-day follow-up period. After intradermal allergy testing, participants with negative allergy test results who satisfied the study inclusion criteria received treatment with either the trial filler or the control filler in the nasolabial folds on both sides of the face. The participants were at least 18 years of age and were in good health. Female participants were required to be non-pregnant. Please refer to NCT03844529 in ClinicalTrials.gov for the detailed inclusion and exclusion criteria. Each participant enrolled in this trial was followed-up for adverse events for 52 weeks after treatment. The injection sites were observed for 21 days post-treatment, and the injection site symptoms were also recorded as adverse events. Immunological examinations were performed at screening and then at 4 and 24 weeks post-treatment.

This study was approved by the Institutional Review Boards of Tri-Service General Hospital (Taipei, Taiwan; IRB No. 1-107-03-001; date of approval: 21 September 2018), Chang Gung Medical Foundation (Taipei, Taiwan; IRB No. 201801242A0; date of approval: 30 August 2018), and the Research Ethics Committee of National Taiwan University Hospital (Taipei, Taiwan; REC No. 201809007DSA; date of approval: 2 October 2018). All participants signed the informed consent approved by the IRB at each institute. This study is registered with ClinicalTrials.gov (NCT03844529).

### 2.3. Skin Allergy Testing

Participants underwent intradermal allergy testing with either the investigational device or the comparator device. The same device was used for subsequent injection treatment. Participants were asked to observe the test site for symptoms such as erythema, tenderness, swelling, and itching lasting for more than 24 h, and were required to return to the doctor on the 7th day (+3 days) after the test to evaluate whether they are allergic to the given injection. The allergic reactions were classified as mild, moderate, or severe [7].

Among the 279 recruited participants, skin allergy test results were evaluated by study doctors for 256 participants. The other 23 participants were deemed screen failures before evaluation, and none of them reported any discomfort.

### 2.4. Immunological Tests

Immunological tests were performed at screening and then at 4 and 24 weeks after treatment. The tests included IgG, IgM, C3, and C4. Turbidimetry was used to examine the quantity of IgG, IgM, C3, and C4 in the serum. The results were judged as normal or abnormal according to the integrated standard. The normal ranges were defined as follows: (1) IgG: 800–1600 mg/dL; (2) IgM: 40–230 mg/dL; (3) C3: 90–180 mg/dL; and (4) 10–40 mg/dL.

### 2.5. Statistical Analysis

The baseline demographics and injection doses in the test and control groups were compared using a two-sample *t*-test, Mann–Whitney U test, or Chi-square test, depending on the data type and distribution. The *p*-values were also provided. The number of hypersensitivity reactions was recorded, and the incidence rate was calculated. Fisher’s Exact test was used to compare the difference between groups, with *p*-values provided. All statistical tests were performed with a significance level of 0.05 (two-tailed test).

## 3. Results

A total of 279 volunteers were recruited in this trial and 252 participants successfully completed the screening process. All 252 participants met the inclusion and exclusion criteria and tested negative in the skin allergy test. Treatment was administered to 123 FULLSGEN participants and 129 FACIALGAIN participants. During the trial, two participants from the test group and five participants from the control group withdrew their consent and exited the trial early, resulting in a total of 245 participants who completed the trial.

Baseline demographic data and average injection doses for the 252 study participants are shown in Table 1. There were no significant between-group differences for the FULLSGEN and FACIALGAIN study populations, with mean ages of 45.0 and 44.9 years, respectively. The majority of each group were female (94.3% and 93.0%, respectively). The mean height and body weight values were similar between the groups, as were BMI values (FULLSGEN: 23.0 kg/m^2^, FACIALGAIN: 23.2 kg/m^2^) and average injection doses (1.9 mL and 2.1 mL, respectively).

### 3.1. Hypersensitivity Reaction

Of the 256 evaluated participants, there were no positive skin allergy results in the FULLSGEN group (0/124, 0.0%), whereas two FACIALGAIN recipients had positive results (2/132, 1.5%). There was no significant difference in the positive rate of the skin allergy test between the two groups (Table 2). At the end of this study, only one FULLSGEN recipient had a positive result (1/123, 0.81%), and there were no hypersensitivity reactions in the FACIALGAIN group (Table 2). The participant with the hypersensitivity reaction recovered without sequelae within 1 day.

### 3.2. Immunological Data

To detect changes in immunoglobulins and complements, samples from the 123 participants in the test group and the 129 participants in the control group were studied. No significant between-group differences were observed for the immunological test data at any of the study visits (*p* < 0.05). Similarly, changes between visits in the turbidimetry results for all four immunoglobulins (IgG, IgM, C3, and C4) were acceptable and are summarized in Table 3. During the 24 weeks, the observed differences in the turbidimetry results had no effect on clinical safety, and none of the study participants developed significant antibodies against porcine Type I collagen. No untoward clinical symptoms were recorded at the test sites. Importantly, participants did not display any unusual incidence or characteristics of adverse events or any evidence of clinical symptoms up to 52 weeks.

The IgG results in Table 3 are representative of those observed for all four antibody classes. The majority of the study participants did not show changes in demonstrable antibodies to collagen during the 24 weeks. A total of 92.7%, 94.3%, and 95.1% of test group participants were judged as normal separately at study entry, the 4th week, and the 24th week. On the other hand, 93.0%, 92.2%, and 93.0% of control group participants were judged as normal separately at study entry, the 4th week, and the 24th week. Table 3 provides the immunological test results for all 252 study participants administered study treatment.

The C3 results showed that a certain number of people had values lower than the normal range during the 24 weeks. A total of 30.1%, 26.0%, and 16.3% of test group participants had lower values separately at study entry, the 4th week, and the 24th week. On the other hand, 21.7%, 23.3%, and 14.7% of control group participants had lower values separately at study entry, the 4th week, and the 24th week.

The C4 results showed that the majority of participants were judged as normal. A total of 93.5%, 93.5%, and 95.1% of test group participants were judged as normal separately at study entry, the 4th week, and the 24th week. On the other hand, 93.8%, 93.8%, and 93.8% of control group participants were judged as normal separately at study entry, the 4th week, and the 24th week. A total of 6.5%, 6.5%, and 4.1% of test group participants and 6.2%, 6.2%, and 5.4% of control group participants had values that were a little lower or higher than the normal range, considered borderline but still acceptable as normal. The C3 and C4 results showed that neither the test group nor the control group experienced any reductions during the 24 weeks.

### 3.3. Adverse Event Profiles

The participants did not exhibit any unusual incidence or adverse event profile, nor evidence of any clinical symptoms prior to study completion. Importantly, participants did not exhibit any unusual incidence or adverse event profile or evidence of any clinical symptoms prior to study completion. Three severe adverse events were recorded throughout the trial, including cellulitis of the lower right side of the face, a bilateral ovarian teratoma, and gallstones with chronic cholecystitis. All were irrelevant to the device, and all cases recovered without sequelae. Thus, no serious adverse events were considered related to the study implants.

The proportion of participants with adverse events related to the device were quite similar in the test group (82.1%) and the control group (74.4%), with no significant difference (*p* = 0.139). Most related adverse events were classified as mild, were mostly predictable, and only required observation or drug treatment. The majority of these related adverse events continued for no more than 30 days in both the test group (95%) and the control group (95.5%). Almost all the related adverse events were resolved without sequelae in both the test group (98.8%) and the control group (99.7%). Most adverse events were classified as common complications associated with dermal filler treatment, occurring around the injection site. Five related adverse events were still present but stable at 52 weeks. Four of these adverse events were nodules occurring in the test group, whereas the other was urticaria in the control group. The participant with urticaria, after being diagnosed with an adverse reaction caused by specific food allergens, significantly improved symptoms by managing and controlling the allergens in their diet. Although the urticaria event was categorized as “possible” (related adverse event), there was no evidence supporting a causal relationship between the event and the dermal injectable collagen.

One FULLSGEN recipient (0.8%) experienced a mild allergic reaction on the treatment day but recovered without any lasting complications. The proportion of participants who felt pain around the injection site was higher in the test group (43.1%) than in the control group (29.5%), with a statistically significant difference (*p* = 0.024). In addition, the pain was mild in both groups, and the prognosis for the subjects was good without sequelae. Furthermore, there was no significant difference in VAS and TPS scores between the two groups, suggesting that although the incidence of pain was significantly higher in the test group than in the control group, it did not affect safety.

## 4. Discussion

Collagen-based injectable products are extensively utilized in clinical settings for aesthetic and regenerative surgery. However, despite their widespread use, concerns regarding allergic reactions and adverse events remain significant over time [6]. For example, in a 1983 investigation involving approximately 7000 Zyderm implant recipients, intradermal allergy testing revealed an incidence rate of 3% for positive test site reactions [4]. Interestingly, among participants with negative test site reactions in that intradermal allergy testing, there was still a 1.5% incidence rate of allergic site reactions when treated with bovine collagen [4]. These findings indicate that a 1.5% incidence of allergic reactions to collagen therapy is considered an acceptable risk based on research conducted with the Zyderm device. This suggests that despite negative intradermal allergy test results, allergic reactions may still occur during collagen treatment. This information underscores the importance of comprehensive allergy screening and monitoring for collagen-based therapies to ensure patient safety. During a 12-month follow-up period in another study, improvement in the Wrinkle-Severity Rating Scale score was slightly higher in the porcine collagen filler (TheraFill^®^) group than the bovine collagen filler (KOKEN^®^) group, although the difference was not statistically significant [8]. No serious device-related adverse events were observed, and both materials were tolerable in most cases. The study researchers concluded that the long-term effects of porcine collagen may be a good alternative to bovine collagen filler [8].

In this trial, both study implant devices were composed of porcine dermal collagen, which can induce allergic reactions in some individuals. To mitigate this risk, intradermal allergy testing was conducted during the screening phase to identify participants who may be allergic to this type of collagen. Participants who tested positive for allergies were excluded from receiving treatment with the device. Nevertheless, one recipient treated with the novel cross-linked porcine-based dermal injectable collagen Sunmax FULLSGEN experienced a mild allergic reaction after receiving the treatment, resulting in an incidence rate of 0.8% (1/123). The participant recovered on the same day without any lasting complications. Notably, none of the 129 recipients treated with commercially available Sunmax FACIALGAIN experienced allergic reactions following treatment.

Intradermal allergy testing of FULLSGEN recipients yielded no positive results (0/124). After treatment, 0.8% (1/123) of participants with negative results from the intradermal allergy test developed mild allergies during treatment. These participants experienced a swift recovery without any lasting complications. In the FACIALGAIN group, the positive rate of intradermal allergy testing was 1.5% (2/132). Therefore, this trial indicates minimal potential for allergic reactions for both dermal injectable collagens. A 2007 study that investigated the hypersensitivity incidence of a cross-linked porcine collagen implant used intradermally for correcting rhytids and scars recorded very low potential for hypersensitivity (0.58%), suggesting that intradermal skin testing is unnecessary before using the implant [7].

This safety evaluation focused on participants’ adverse events and device-related adverse events. No serious device-related adverse events were observed during the trial. In both study groups, most device-related adverse events were classified as mild, were mostly predictable, and only required observation or drug treatment. A significant portion of the device-related adverse events resolved within a time frame of 0 to 30 days.

When comparing the between-group incidence rate of device-related adverse events, there was no significant difference overall (*p* = 0.139). One notable exception was the significantly higher incidence of pain at the injection site among the FULLSGEN recipients compared with the FACIALGAIN recipients (43.1% vs. 29.5%; *p* = 0.024), although the severity of pain experienced in both groups was classified as mild. The prognosis for participants in both groups was favorable, with no lasting complications or sequelae observed. These findings indicate that despite the higher incidence of pain with FULLSGEN administration, this did not adversely influence the overall safety of the trial.

It has been indicated that porcine collagen does not cause much of an allergic response because it is almost similar to human collagen [14]. Consequently, as the investigational device consists of animal-sourced material, it is possible that a small number of people are allergic to porcine collagen and will experience an allergic reaction (an incidence rate of 0.8%). However, participants in the trial were not at higher risk compared to persons receiving conventional dermal filler treatments, and this study carefully controlled these risks. Firstly, the injection site was cleaned, disinfected, and massaged after injection to ensure the even distribution of collagen. Secondly, in the event of any adverse events during treatment, study physicians were instructed to provide appropriate treatment. All the measures above were implemented to ensure safety and minimize the impact on participants.

This study was conducted in Taiwan, with a predominantly Asian ethnic composition. Caucasian, Latino, African, and other ethnic groups were not included in this evaluation of skin and immunological responses. However, there are differences in dermal thickness between ethnic groups. Therefore, it would be interesting to see how well the devices perform across different ethnic groups. Additionally, this study did not include gender identity, which means this study cannot account for any potential associations between non-cisgender classifications and dermal thickness.

In this trial, both study devices exhibited an acceptable incidence rate of allergy, with the investigational device showing a lower allergy rate compared to the comparator. Furthermore, both devices are composed of purified porcine atelocollagen, and the removal of telopeptides can reduce the likelihood of allergic reactions [15,16]. A number of research articles demonstrate that the cross-linking process lowers the allergy rate of collagen materials when used in the human body [17,18]. In this clinical trial, the investigational device FULLSGEN did not induce immunological responses and had a similar allergy rate compared to the marketed product (1.3%) [5]. Consequently, although both devices are made of porcine collagen, intradermal allergy testing prior to treatment appears to be unnecessary.

## 5. Conclusions

FULLSGEN is the latest generation of porcine-based dermal injectable collagen developed by Sunmax Biotechnology, featuring a patented cross-linking technology. This clinical trial reported no serious device-related adverse events, which indicates a favorable safety profile for both study devices. Most device-related adverse events were anticipated and categorized as mild in severity. Importantly, these device-related adverse events resolved within a period of 0 to 30 days post-injection, without any long-lasting or permanent complications or sequelae. Skin allergy testing revealed a low incidence of hypersensitivity, and immunological testing revealed no safety concerns for either device.

In summary, the trial results demonstrate that the investigational device, FULLSGEN, has an even lower potential for hypersensitivity than the comparator device (FACIALGAIN), supporting the safe use of FULLSGEN for the correction of nasolabial folds without the need for pretreatment skin testing.

## Figures and Tables

**Table 1:** Subject demographics at baseline and injection doses for the 252 study participants.

Characteristics	FULLSGEN Group (n = 123)	FACIALGAIN Group (n = 129)	*p*-Value
Age (years)			
Mean ± STD	45.0 ± 8.58	44.9 ± 7.63	0.872 ^a^
Sex			
Male (n, %)	7 (5.7)	9 (7.0)	
Female (n, %)	116 (94.3)	120 (93.0)	0.676 ^c^
Height (cm)			
Mean ± STD	160.6 ± 6.84	159.8 ± 6.49	0.334 ^b^
Weight (kg)			
Mean ± STD	59.5 ± 11.63	59.6 ± 11.54	0.870 ^b^
BMI (kg/m^2^)			
Mean ± STD	23.0 ± 3.86	23.2 ± 3.60	0.614 ^b^
Injection dose (mL)			
Mean ± STD	1.9 ± 0.74	2.1 ± 0.73	0.108 ^b^

^a^ two-sample *t*-test, ^b^ Mann–Whitney U test, ^c^ Chi-square test.

**Table 2:** Hypersensitivity reactions after injection, including skin allergy testing and treatment.

	Number	Population	Incidence	*p*-Value
Skin Allergy Test				
FULLSGEN group	0	124	0.00%	
FACIALGAIN group	2	132	1.52%	0.499 ^a^
After Treatment (within 1 week)				
FULLSGEN group	1	123	0.81%	
FACIALGAIN group	0	129	0.00%	NA ^b^

^a^ Fisher’s Exact test, ^b^ not applicable.

**Table 3:** Immunological test results for all 252 study participants administered study treatment.

		FULLSGEN Group (N = 123)						FACIALGAIN Group (N = 129)					
		Visit 1		Visit 3		Visit 5		Visit 1		Visit 3		Visit 5	
		(day −14 to 1)		(week 4 ± 7 days)		(week 24 ± 7 days)		(day −14 to 1)		(week 4 ± 7 days)		(week 24 ± 7 days)	
IgG (mg/dL)		N	%	N	%	N	%	N	%	N	%	N	%
	Normal (800~1600 mg/dL)	114	92.7%	116	94.3%	117	95.12%	120	93.02%	119	92.2%	120	93.0%
	Higher (>1600 mg/dL)	8	6.5%	6	4.9%	5	4.07%	8	6.20%	10	7.8%	9	7.0%
	Lower (<800 mg/dL)	1	0.8%	1	0.8%	1	0.81%	1	0.78%	0	0.0%	0	0.0%
IgM (mg/dL)													
	Normal (40~230 mg/dL)	114	92.7%	120	97.6%	119	96.75%	126	97.67%	120	93.0%	121	93.8%
	Higher (>230 mg/dL)	6	4.9%	1	0.8%	2	1.63%	1	0.78%	6	4.7%	5	3.9%
	Lower (<40 mg/dL)	3	2.4%	2	1.6%	2	1.63%	2	1.55%	3	2.3%	3	2.3%
C3 (mg/dL)													
	Normal (90~180 mg/dL)	86	69.9%	91	74.0%	103	83.74%	101	78.29%	99	76.7%	110	85.3%
	Lower (<90 mg/dL)	37	30.1%	32	26.0%	20	16.26%	28	21.71%	30	23.3%	19	14.7%
C4 (mg/dL)													
	Normal (10~40 mg/dL)	115	93.5%	115	93.5%	117	95.12%	121	93.80%	121	93.8%	121	93.8%
	* Borderline (10~40 mg/dL)	8	6.5%	8	6.5%	5	4.07%	8	6.20%	8	6.2%	7	5.4%
	Higher (>40 mg/dL)	0	0.0%	0	0.0%	1	0.81%	0	0.00%	0	0.0%	1	0.8%

* As the definitions of normal ranges for laboratory test data differed by hospital, about 4.05~6.07% fell outside the study-defined range of normal values.

## Data Availability

All data generated or analyzed during this study are included in this published article.
